# An unknown apocarotenoid signal alters plant development by modulating shoot and root apical meristem activities

**DOI:** 10.1093/plphys/kiaf448

**Published:** 2025-09-26

**Authors:** James M Bradley

**Affiliations:** Assistant Features Editor, Plant Physiology, American Society of Plant Biologists; Department of Cell and Systems Biology, University of Toronto, Toronto, ON M5S 3B2, Canada

The plant body plan is directed via organogenesis from stem cell niches in the shoot and root apical meristems. The process of organogenesis is plastic and can be modulated by abiotic cues and endogenous signals to allow the plant to produce new organs that are appropriately acclimated to their environment (Bar and Ori 2014). Endogenous signals are also vital to coordinate the differentiation of specialized cell types and their plastids (Sierra et al. 2023). For example, during the process of leaf development, proplastids must commit to differentiate into chloroplasts to conduct photosynthesis. In the columella cells of the root, proplastids differentiate into starch-accumulating amyloplasts to contribute to gravitropic responses (Sierra et al. 2023). Yet many plastid-resident proteins are encoded in the nuclear genome and must be imported during the process of plastid differentiation (Woodson and Chory 2008). There must therefore be signals that originate from the plastids and transmit information to the nucleus to coordinate plastid developmental status with nuclear gene expression (Woodson and Chory 2008; Sierra et al. 2022, 2023).

Carotenoids are plastid-derived orange, yellow, or red pigments present in all photosynthetic organisms that primarily serve photoprotective roles in chloroplasts (Nisar et al. 2015). These molecules are also responsible for the colors of flowers and fruits and are derived into an array of secondary metabolites, collectively called apocarotenoids (Nisar et al. 2015; Hou et al. 2016). Since carotenoids are such an important constituent of chloroplasts, it is perhaps unsurprising that their apocarotenoid derivatives have been proposed as signals to communicate information between the plastids and the nucleus during leaf development (Sierra et al. 2023). Indeed, some apocarotenoids are already well known to regulate plant growth and development. For example, the hormones abscisic acid and strigolactone are apocarotenoid derivatives regulating diverse aspects of plant development from shoot branching to germination (Umehara et al. 2008; Toh et al. 2012; Nisar et al. 2015). Besides these well-characterized examples, there are predicted to be many uncharacterized apocarotenoid small molecules that play roles in plant development and signaling with plant-associated organisms (Moreno et al. 2021).

In the last decade, genetic evidence has emerged for an unknown apocarotenoid signal that coordinates leaf development with chloroplast biogenesis (Hou et al. 2016; Fig. A). In 2014, Avendaño-Vázquez et al. became interested in a mutant called chloroplast biogenesis 5 (*clb5*) that arrests proplastid-to-chloroplast development early during differentiation. The *clb5* homozygote has an albino phenotype that maps to a mutation in the *ZDS* gene involved in carotenoid biosynthesis. This mutant also has a striking leaf defect in which radially symmetric needle-like leaves form in lieu of wild type laminar leaves. This developmental defect is specific to *clb5*, as other loss-of-function carotenoid biosynthetic albino mutants, such as *pds3*, display laminar leaves. Neither abscisic acid nor strigolactones, both derived from carotenoids, rescue the *clb5* leaf defect, indicating that the defect is not due to deficiencies of these hormones (Avendaño-Vázquez et al. 2014). However, genetic ablation of *PDS*, encoding the biosynthetic enzyme upstream of *ZDS*, could rescue the leaf defect, suggesting that the unknown apocarotenoid signal (which they called ACS1) is derived from *cis*-carotenoids that accumulate upstream of *ZDS* and below *PDS* (Avendaño-Vázquez et al. 2014; Hou et al. 2016). Further epistasis analysis between *clb5* and plastid-localized carotenoid cleavage dioxygenase (*CCD*) genes identified the *clb5 ccd4* double mutants that could partially rescue leaf shape, indicating that CCD4 promotes ACS1 synthesis (Avendaño-Vázquez et al. 2014). The leaf defect could also be rescued by reducing light fluency, suggesting that light promotes ACS1 accumulation (Escobar-Tovar et al. 2021). Indeed, light has been implicated in the nonenzymatic production of other apocarotenoids, such as β-ionone (Hou et al. 2016). The *clb5* mutant also shows strong downregulation of nuclear-encoded genes required for correct chloroplast biogenesis (Hou et al. 2016), suggesting that ACS1 acts as a retrograde signal communicating information from the plastid to the nuclear genome and coordinating plastid-to-chloroplast differentiation with proper leaf development.

**Figure.**
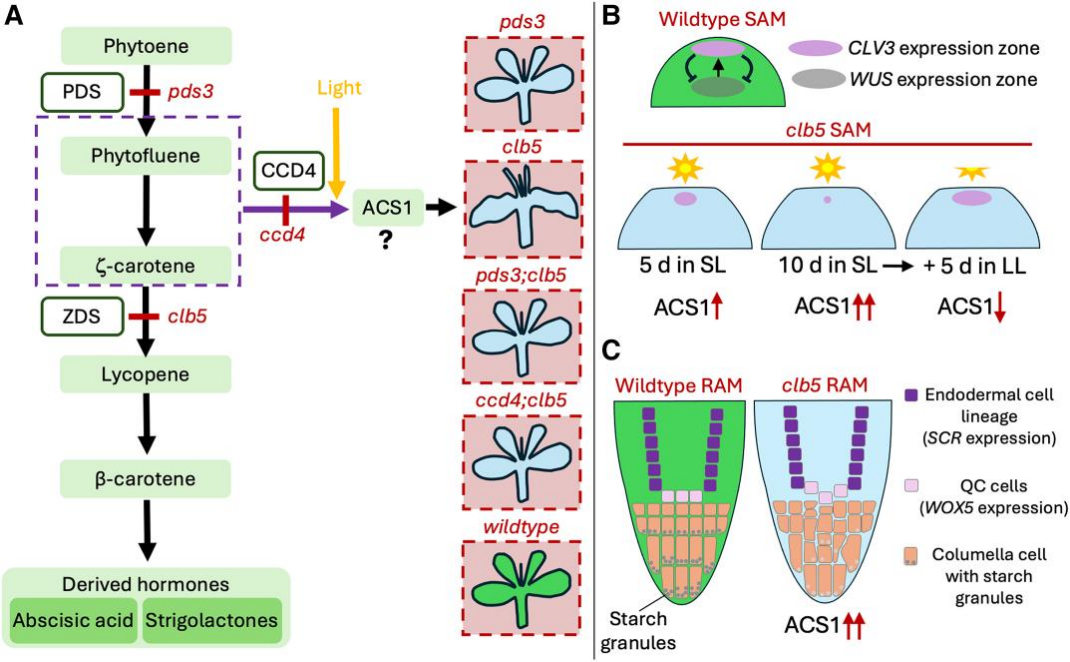
**A)** Genetic analysis of mutants in the carotenoid biosynthesis pathway suggests an unknown apocarotenoid signal, called ACS1. ACS1 is predicted to be derived from phytofluene or ζ-carotene via CCD4 and the action of light. Loss of *PDS* (*pds3*) results in an albino phenotype. Loss of *ZDS* (*clb5*) results in radially symmetric needle-like leaves and downward-curved bumpy cotyledons. The *clb5* leaf defects can be suppressed by loss-of-function mutation in *PDS* or *CCD4*. **B)** The SAM is maintained by a negative feedback loop involving the genetic regulators *CLV3* and *WUS*. The *clb5* mutant lacks the typical dome shape of the SAM and lacks *WUS* expression. *CLV3* expression was detected in *clb5* seedlings, but the levels diminish with time spent under SL conditions. Upon shifting to LL conditions, *CLV3* expression recovers, presumably associated with reduced ACS1 levels. **C)** In the RAM, a small cluster of QC cells (pink) maintains surrounding stem cell initials in a pluripotent state. Two important regulators of the QC are *SCR*, expressed in the endodermal cell lineage (purple), and *WOX5*, expressed in the QC cells. In *clb5*, abnormalities were found in the organization of the QC cells and columella cells (orange), and this was coincident with reduced starch accumulation. CCD4, carotenoid cleavage dioxygenase 4; PDS, phytoene desaturase; ZDS, ζ-carotene desaturase.

In this issue of *Plant Physiology*, Sierra et al. (2025) further interrogate the developmental defects induced by ACS1 in the *clb5* mutant. Unlike previous studies with *clb5*, here the authors used light as a tool to modulate ACS1 accumulation. By shifting plants from standard light (SL; high ACS1) to low light (LL; low ACS1), the authors could infer the developmental window within which ACS1 acts to affect leaf development. They grew *clb5* and *pds3* (control) plants in SL conditions for increasing periods of time (4–10 d). If left in SL conditions, *clb5* plants developed radially symmetric primary leaves. However, shifting plants to LL after 4 to 5 d in SL resulted in most plants developing laminar leaves, whereas shifting plants after 6 d in SL saw the development of radially symmetric primary leaves. Interestingly, these later-shifted plants developed a second pair of leaves that had acquired a laminar form. The authors concluded that there must be a defined developmental window during which ACS1 affects the fate of cells destined to become leaves, suggesting a potential role for ACS1 in the shoot apical meristem (SAM), where leaf organogenesis occurs.

Upon inspection, the authors indeed noted an irregular SAM in *clb5*, which lacked the typical dome shape of wild type. The SAM is maintained through a negative regulatory loop involving the small secreted peptide CLAVATA3 (CLV3) and the transcription factor WUSCHEL (WUS). *WUS* is expressed in the SAM organizing center, a region just below the stem cell niche at the shoot apex, and is a positive regulator of *CLV3* expression, which itself negatively regulates *WUS* and stem cell accumulation (Clark et al. 1995; Laux et al. 1996; Fletcher 2018; Fig. B). To investigate SAM organization, Sierra et al. (2025) introduced *pCLV3::CLV3-GUS* and *pWUS::GUS* reporters into wild type and *clb5* backgrounds. They did not observe *WUS* expression in the *clb5* mutant, even after the emergence of the second leaf pair. However, *CLV3* expression was clearly seen in 5-d-old seedlings and decreased over time under SL conditions so that by day 10 expression was very low. This suggested that the accumulation of ACS1 leads to a depletion of the stem cell population in *clb5*. The absence of *WUS* expression in *clb5* is consistent with the finding that WUS is not required to establish the SAM stem cell population but is required to maintain meristem integrity during growth (Laux et al. 1996; Zhang et al. 2017). The authors then switched their SL-grown 10-d-old *clb5* plants to LL for a further 5 d and found that *CLV3* expression recovered, and this correlated with the development of laminar leaves. Given the dysregulation of the *CLV3*-*WUS* module in the SL-grown *clb5* mutant, the authors thus concluded that ACS1 disrupts SAM integrity. However, since shifting to LL rescued *CLV3* expression, the authors suggested that ACS1 causes a reversible developmental effect rather than a toxic by-product that irreversibly damages plant development.

The authors then turned their attention to below-ground effects and assessed the role that ACS1 might play in root apical meristem (RAM) organization and function. The RAM consists of distinct stem cell initials maintained adjacent to rarely dividing quiescent centre (QC) cells (Sarkar et al. 2007; Dubrovsky and Vissenberg 2021). The stem cell initials divide asymmetrically, with the daughter cells more distal from the QC cells going on to differentiate into various cell types of the root (Petricka et al. 2012; Dubrovsky and Vissenberg 2021). The stem cell initials just below the QC cells, for example, give rise to differentiated columella cells (Petricka et al. 2012). Just as in the SAM, small peptide ligands and transcription factors are involved in maintaining stem cell niches in the root (Petricka et al. 2012). One key transcription factor is WUSCHEL-related homeobox 5 (WOX5). This protein is expressed in QC cells and non–cell autonomously maintains the pluripotency of surrounding stem cells (Petricka et al. 2012). For example, in *wox5* mutants, columella stem cell initials begin to differentiate and acquire starch granules (Sarkar et al. 2007). Another important regulator of the RAM is the GRAS transcription factor scarecrow (SCR), which is required for QC specification and asymmetric division of surrounding stem cells (Petricka et al. 2012).

Accordingly, Sierra et al. (2025) used *proWOX5::GFP* and *proSCR::SCR-GFP* reporters to characterize the RAM of *clb5* plants. Analysis of 5- and 10-d-old plants grown in SL showed no clear differences in expression patterns from the *WOX* or *SCR* promoters, as compared with *pds3* or wild type plants. However, the authors did note misaligned QC cells and irregular divisions among the columella cells, suggestive of additional stem cell divisions (Fig. C). The same was not true for stem cell initials above the QC that go on to differentiate into the vasculature or epidermal cell layers—these appeared normal. The columella defects prompted closer investigation. During columella cell differentiation, the cells acquire starch-containing amyloplasts, which are important for graviperception and gravitropism (Sierra et al. 2023). Upon staining roots, Sierra et al. (2025) observed reduced starch levels in the *clb5* columella cells as compared with *pds3* and wild type lines, suggesting that *clb5* might be less able to sense gravity. Indeed, when they rotated plants by 90°, the roots of *clb5* plants were unable to fully adjust their root tips to account for the change in gravity as compared with *pds3* or wild type plants. Despite this, Sierra et al. (2025) did detect some starch in *clb5*, suggesting that amyloplasts had successfully differentiated in the columella cells, unlike the plastids located in leaves that were completely unable to differentiate into chloroplasts.

In sum, Sierra et al. (2025) have further probed the developmental consequences of overproduction of the unknown small molecule ACS1 in the *clb5* background. Specifically, they show that high ACS1 levels, which are associated with SL conditions, affect shoot and root meristem organization. Using shift experiments from SL to LL, the authors demonstrated that ACS1 is not a toxic product that causes irreversible developmental damage but rather a dynamic signal that is reversible and acts during a defined developmental window to shape leaf organogenesis. For wild type, the implication is that environmental and metabolic cues may be “read” by the plastids (where ACS1 is assumed to be produced) and communicated to the nucleus by fine-tuning ACS1 levels. This in turn would affect the shoot and root meristem activities, leading to an altered plant body plan more suited to the environment. Of course, the burning questions remain. First, what is the nature of ACS1? Second, what are the direct targets of ACS1? To answer the latter, forward genetics could prove useful to identify mutants that suppress the *clb5* radially symmetric leaves; at least a subset of suppressors would be direct ACS1 targets.

## Data Availability

No new data were generated or analyzed in support of this research.
